# Evidence of pial synagiosis through ventriculoperitoneal shunt entry site in a patient who manifested Moyamoya syndrome later in childhood: A case report and historical perspective

**DOI:** 10.1007/s00381-023-06263-x

**Published:** 2024-01-05

**Authors:** Awn AlEssa, Alaa AlSahli, Tariq AlJared

**Affiliations:** 1 Neurosurgery Department, King Abdullah Specialized Children Hospital, Riyadh, Saudi Arabia; 2 College of Medicine, King Saud Bin Abdulaziz University for Health Sciences, Riyadh, Saudi Arabia; 3 King Abdullah International Medical Research Center, Riyadh, Saudi Arabia

**Keywords:** Moyamoya syndrome, Revascularization, Burr hole, Ventriculoperitoneal shunt

## Abstract

The term Moyamoya , or “puff of smoke” in Japanese , was first used in 1969 by Suzuki and Takaku to describe the classical appearance of collateral blood vessels in response to progressive vascular stenosis of distal internal carotid artery (ICA). Such condition may result in various clinical presentations ranging from strokes to developmental delays. In order to cease the progression of such stenotic vasculopathy, several means of revascularization have been developed over the years. In this paper we present a case of a two-year-old girl with history of myelomeningocele repair and ventriculoperitoneal shunt insertion followed by manifestation of Moyamoya syndrome later in childhood as an evidence of revascularization through a burr hole. To our knowledge, this paper is the first of its kind to report such findings in one patient. Moreover, this paper provides a historical perspective on the development of different types of revascularization techniques.

## Introduction

Moyamoya disease comprises idiopathic progressive vascular stenosis of circle of Willis commonly at the level of distal internal carotid artery (ICA) [1]. The term Moyamoya, or “puff of smoke” in Japanese, was first used in 1969 by Suzuki and Takaku to describe the classical appearance of collateral blood vessels forming in close proximity to the base of the skull as a direct consequence of a disease entity later known as Moyamoya disease (MMD) [2]. Cerebrovascular stenosis may occur in parallel with systemic conditions giving rise to Moyamoya syndrome (MMS). Clinical features of this entity are unique in the fact that vascular stenosis is bilateral in nature and may progress to complete occlusion. In the last decade, the prevalence and incidence of MMD in Japan have increased to reach 10.5/100,000 and 0.94/100,000, respectively [3]. Clinical features of MMD may vary according to the age of the patient. However, major symptoms are product of cerebral hypoperfusion. Patients may present with transient ischemic attack (TIA), ischemic or hemorrhagic strokes, seizures, and cognitive impairment [4]. With advancement in surgical techniques, myriad of modalities have been developed throughout history. Direct, indirect, and combined revascularization techniques can be used to bypass occluded vessels. All aiming to surgically induce pial synagiosis and external carotid—internal carotid circulation formation. In this report, we share a unique presentation of MMS in parallel with myelomeningocele (MMC) and sickle cell disease (SCD).

## Case report

**Fig. 1:**
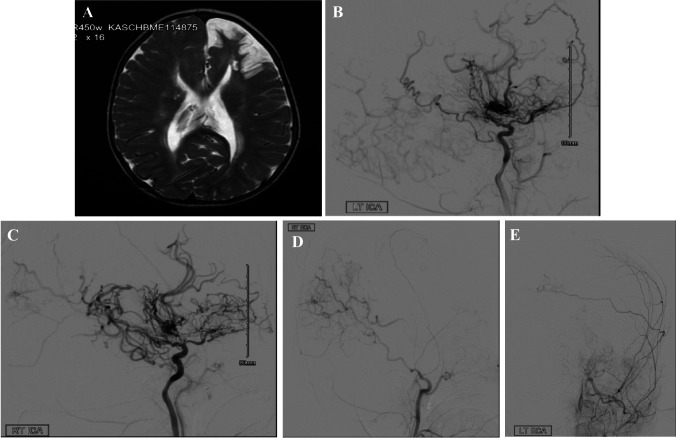
**A** T2 MRI imaging showing left frontal lobe ischemic insult and atrophy. **B** Cerebral angiography of left ICA showing stenosis of ICA and collateral vessel formation. **C** Cerebral angiography of right ICA showing marked collateral vessels of leptomeningeal and lenticulostriate source. **D** Right ECA (occipital artery) vascularization through the shunt burr hole. **E** Left ECA (occipital artery), crossing to the contralateral side to vascularize through the shunt burr hole

We are reporting a rare case of 2-year-old girl who is known case of myelomeningocele (MMC) and hydrocephalus. At 1 month of age, she underwent repair and a contour-valve of ultra small size ventriculoperitoneal shunt insertion for MMC and hydrocephalus, respectively. Patient has been paraplegic since birth. She was due for her regular follow-up and imaging in first of year of life, but parents were not complaint. Unfortunately, patient has presented to the clinic after 1 year where the mother reported left hand weakness and right-hand preference. Magnetic resonance imaging (MRI) of the brain and spine were done urgently and showed left frontal lobe ischemic insult and atrophy (Fig. 1A).

After elaborating more in the history, we found her both parents have sickle cell disease trait with strong family history of SCD. Hemoglobin electrophoresis was performed for the patient confirming she has SCD. Magnetic resonance angiography (MRA) was requested to rule out Moyamoya syndrome; it showed bilateral occlusion of internal carotid artery (ICA) at supraclinoid segments level and associated with multiple tortuous collaterals. Left anterior cerebral artery (ACA) was found to be smaller in caliber compared to right counterpart. However, both are patent. Nevertheless, high signal intensity was noted on FLAIR MRI at cortical sulci indicating collaterals.

Cerebral angiography wad done and showed bilateral steno-occlusive ICAs and both middle cerebral arteries (MCA) M1 segments with prominent lenticulostriate and leptomeningeal collaterals mainly from right side confirming the diagnosis of MMS in this patient (Fig. 1B, C).

Interestingly, significant collaterals vessels formed from right occipital artery directly to posterior circulation through the burr hole opening of the ventriculoperitoneal shunt, helping to replenish the right hemisphere (Fig. 1D).

This burr hole also recruited supply from the left occipital artery (contralateral side). This has protected the right hemisphere relative to the insufficiency encountered by the left hemisphere (although disease is bilateral) (Fig. 1E).

The used modalities for vasculature evaluation in this patient was part of careful preoperative planning to minimize the risk of collateral vessel disruption. During surgery, we utilized the right superficial temporal artery (STA) for revascularization, thus maintaining any replenishment for MMD affected areas.

Encephaloduroarteriosynangiosis (EDAS) surgery was offered to family which was planned into 2 stages, but unfortunately, they refused. Therefore, serial follow-up visits were given in our clinic as well as hematology’s to optimize her SCD. It is important to highlight that she never had vaso-occlusive crisis, and all strokes had occurred in MMS occluded territory. During her follow-up with brain MRI, results showed multiple lacunar infarctions, which indicate ongoing silent strokes. Clinically, she also was noticed to have delays in speech and microcephaly likely attributed to ongoing ischemia. Patient underwent right EDAS surgery 1 year after the onset of left-hand weakness and the establishment of diagnosis as MMS. After the surgery, patient’s left hand function improved from movement against gravity to partial handling of objects. Then, she underwent left side EDAS surgery at later stage.

## Discussion

Moyamoya disease is a cerebrovascular arteriopathy of unknown origin characterized by progressive stenosis and, ultimately, occlusion of the distal intracranial internal carotid arteries (ICA) and the proximal branches of the anterior and middle cerebral arteries [5]. The progressive occlusion of supraclinoid ICA and its proximal branches, mainly middle cerebral artery and anterior cerebral artery, is seen in the initial phase. As a compensatory phenomenon, collaterals develop from the basal perforators. These collateral vessels appear as “smoky puffs” in carotid angiography [6].

Moyamoya disease accounts for one fifth of the identifiable cerebral arteriopathies in childhood stroke and the most common cerebrovascular disease in children in East Asia [7, 8]. In children, unilateral involvement occurs about 18% and might progress to bilateral involvement within 2 years [9]. If left untreated, the natural history of untreated cases ranges from a slow progression with intermittent events, to rapid neurological deterioration that leads to cognitive decline.

The overall mortality rates in the Japanese literature is as high as 4.3% [10]. The long-term outcome of Moyamoya disease is poor due to inevitable progression that occurs in the majority of patients, with up to 66% having symptomatic progression over a 5-year period following diagnosis [11]. On the other hand, various studies have shown a significant impact on long-term outcome in those patients undergoing surgical revascularization.

Revascularization surgery for symptomatic MMD is considered the standard treatment for preventing further strokes [12]. Moreover, the main objective of surgery is to augment intracranial blood flow. This phenomenon is a natural adaption process to compensate for stenosis of the internal carotid artery (ICA) and it can be achieved by either extracranial-intracranial bypass or vasculogenesis using indirect pial synangiosis [12]. Surgical revascularizations to prevent ischemic stroke is an effective treatment for patients with MMD with an ischemic presentation [13, 14].

This concept has an interesting historical background as most use indirect means of revascularization. Throughout history, certain authors have advocated the use of multiple burr holes as surgical treatment for MMD. The first of whom to propose this technique was Endo et al. in 1989, where he reported 5 pediatric cases of MMD in which frontal cranial burr holes targeting ACA territory were used. Postoperative evaluation via dynamic computed tomography showed shortened mean transit time across ACA territory indicating successful revascularization and preservation of function [15]. Such compelling findings urged surgeons to combine direct and indirect revascularization techniques to augment the effect of both techniques. Suzuki et al. in 1997 combined placement of bilateral frontal cranial burr holes with either STA-MCA anastomosis in children older than 5 years of age or EDAS procedures in those younger than the age of 5. Suzuki et al.’s work was considered a surgical innovation as it was the first to report transposition of frontal branch of STA over cortical surface through a craniotomy. His innovative work was proved effective in reversal of ischemia in 36 pediatric cases [16].

The degree of such collateralization in the Moyamoya patient with hydrocephalus treated by a VP shunt placed via a convexity bur hole has not been previously reported. Endo, Kawanok, Miyasaka, and Yada (1989) noted in a case of Moyamoya disease with intraventricular hemorrhage that a burr hole, made in the frontal region for drainage purposes, induced marked neovascularization, and first published 5 cases with spontaneous revascularization after burr hole procedure in Moyamoya disease. Endo noted in a case of Moyamoya disease with intraventricular hemorrhage that a burr hole, made in the frontal region for drainage purposes, induced marked neovascularization. This procedure involved making a burr hole in both frontal bones and incising both the dura and the arachnoid membrane. Together with conventional surgical therapy for juvenile cases of Moyamoya disease, this operation has been considered beneficial for both the circulation in the frontal region and for the protection of frontal brain function [17]. Singla et al. [18] reported 3 cases with a history of a VP shunt for hydrocephalus and were treated for Moyamoya disease years after their initial shunt procedures. All patients demonstrated spontaneous trans-dural collateral vessels at the ventricular catheter bur hole, as confirmed by angiography during the workup for Moyamoya disease [15, 18].

Although the principle of burr hole procedure has been proved to be effective in case of Moyamoya disease to improve brain vasculature, only few cases show that spontaneous vascularization was found through bur hole of ventriculoperitoneal shunt procedure in patients with Moyamoya disease. These cases are important to be published because the surgeon’s handling of them must be meticulous especially in case of revision.

## Conclusion

We reported this case of hydrocephalus with shunt insertion that unfortunately developed Moyamoya disease later in the life. Understanding of shunt revision in case of Moyamoya disease is crucial for neurosurgeon as those population may carry the potential risks of intracranial hemorrhage occurring during shunt catheter manipulation which might disturb the preexisting brain collaterals coursing through the bur hole site. Alternative options such as altering incisions or placing shunts at new sites while leaving old catheters alone might be helpful in preventing complications. Informed consent should be obtained from families including the potential risk of perioperative stroke when this clinical scenario is encountered.

## Data Availability

Authors declare that data supporting the findings of this study are available within the paper and the supplementary material provided.
